# Improving functional magnetic resonance imaging reproducibility

**DOI:** 10.1186/s13742-015-0055-8

**Published:** 2015-03-31

**Authors:** Cyril Pernet, Jean-Baptiste Poline

**Affiliations:** 1 Centre for Clinical Brain Sciences, Neuroimaging Sciences, University of Edinburgh Chancellor’s Building, 49 Little France Crescent, Edinburgh, EH16 4SB UK; 2 Henry H Wheeler, Jr Brain Imaging Center, Helen Wills Neuroscience Institute, University of California at Berkeley, 3210 Tolman Hall, Berkeley, CA 94720-1650 USA

**Keywords:** Functional MRI, Reproducibility, Scripts, Workflows, Code, Open science

## Abstract

**Background:**

The ability to replicate an entire experiment is crucial to the scientific method. With the development of more and more complex paradigms, and the variety of analysis techniques available, fMRI studies are becoming harder to reproduce.

**Results:**

In this article, we aim to provide practical advice to fMRI researchers not versed in computing, in order to make studies more reproducible. All of these steps require researchers to move towards a more open science, in which all aspects of the experimental method are documented and shared.

**Conclusion:**

Only by sharing experiments, data, metadata, derived data and analysis workflows will neuroimaging establish itself as a true data science.

“Experience has shown the advantage of occasionally rediscussing statistical conclusions, by starting from the same documents as their author. I have begun to think that no one ought to publish biometric results, without lodging a well arranged and well bound manuscript copy of all his data, in some place where it should be accessible, under reasonable restrictions, to those who desire to verify his work.” Galton 1901 [1]

## Introduction

Because current research is based on previous published studies, being able to reproduce an experiment and replicate a result is paramount to scientific progress. The extent to which results agree when performed by different researchers defines this tenet of the scientific method [2,3]. Recently, a number of authors have questioned the validity of many findings in epidemiology or in neuroscience [4,5]. Results can be found by chance (winner’s curse effect), more often in poorly powered studies [6], or be declared significant after too many variations of the analysis procedure [7,8] without controlling appropriately for the overall risk of error (p-hacking effect [6,9]). Additionally, errors in code or in data manipulation are easy to make [10]: it is in general difficult to check for the correctness of neuroimaging analyses. Reproduction is one way to address these issues, given that the probability of a research finding being true increases with the number of reproductions (see Figure two in [4]).

If the reliability of a large proportion of functional magnetic resonance imaging (fMRI) results is questionable, this has serious consequences for our community. Mostly, this means that we are building future work on fragile ground. Therefore we need to ensure the validity of previous results. It is very possible, and some argue likely, that we - as a community - are wasting a large amount of our resources by producing poorly replicable results. We can, however, address the current situation on several fronts. First, at the statistical analysis level, one proposed solution is to be more disciplined and use pre-registration of hypotheses and methods [11]. Providing information about planned analyses and hypotheses being tested is crucial, as it determines the statistical validity of a result, and therefore the likelihood that it will be replicated. This would bring us closer to clinical trial procedures, leading to much more credible results. It does not remove the possibility of analyzing data in an exploratory manner, but in that case p-values should not be attached to the results. Pre-registration is an effective solution to address the growing concern about poor reproducibility, as well as the ‘file drawer’ issue [9,12]. Second, we propose that better procedures and programming tools can improve the current situation greatly. We specifically address this question, because many of the researchers using fMRI have limited programming skills.

Although we aim for reproduction of results with other data and independent analysis methods, the first step is to ensure that results can be replicated within laboratories. This seems an easy task, but it is in fact common that results cannot be replicated after, say, a year or two, when the student or post-doc responsible for the analyses and the data management has left. Increasing our capacity to replicate the data analysis workflow has another crucial aspect: this will allow us to better document our work, and therefore communicate and share it much more easily. It is crucial that we remember that resources are limited, and part of our work is to make it easy for others to check and build upon our findings.

In computer science and related communities, a number of informatics tools and software are available (databases, control version system, virtual machines, etc.) to handle data and code, check results and ensure reproducibility. Neuroscientists working with functional MRI are, however, largely from other communities such as biology, medicine and psychology. Because of the differences in training and the field of research, such informatics tools are not necessarily sufficient, and are certainly not fully accessible to or mastered by all researchers. In this review, we address specifically the community of neuroscientists with little programming experience, and point to a number of tools and practices that can be used today by anyone willing to improve his or her research practices, with a view to better reproducibility. We also recommend observing how other communities are improving their reproducibility. For instance, B Marwick [13] gives an excellent summary of these issues and some solutions for the social sciences, and many of his recommendations may be shared between fields. Improving the capacity of other researchers to reproduce one’s results involves some degree of sharing, through journals, repositories or dedicated websites (Annex 1). These practices, if followed, should be sufficient to allow any researcher to replicate a published fMRI experiment. Here we define replication as the capacity of a colleague to re-execute the analyses on the same dataset [14], but note that this definition varies in the literature [15]. In step 2 below (‘Improving scripts and turning them into workflows’), we expand on good practice for writing and sharing code. Although this can seem daunting for people who do not often write code, our goal is to give some tips to improve everyone’s analysis scripts.

### Reproducible neuroimaging in 5 steps

We define reproducibility as the ability of an entire experiment to be reproduced [16], from data acquisition to results. In some fields, such as computational neuroscience, reproducibility can be readily dissociated from replicability, which is the capacity for exact analytical reproduction of the analysis pipeline, possibly using the same data [14,15]. For fMRI, as for other fields, reproduction is more of a continuum: analytic reproduction (the replication case), direct reproduction (reproducing a result using the same conditions, materials and procedures as in the original publication, but with other subjects), systematic reproduction (trying to obtain the same finding by using many different experimental conditions), and conceptual reproduction (reproducing the existence of a concept using different paradigms). The question we address here is to what extent we can share protocols, data, workflows and analysis code to make fMRI studies easier to replicate and directly reproduce.

### Sharing experimental protocols

Every task-based fMRI study depends on an experimental procedure in which subjects are instructed to passively watch, listen, feel, taste, or smell, or to actively engage in a task. In all cases, stimuli are presented via a computer program that synchronizes with the MRI scanner. Although such procedures are always described in published articles, some details about the order of stimulus presentation, stimulus onset times or stimulus sizes, for example, can be missing. The issue is that such details can determine whether an effect is observed or not. It is therefore paramount to be able to replicate the experimental setup if one wants to reproduce a study. Sharing computer programs (and stimuli) is easily achievable: when publishing an article, the computer program can be made available either as supplementary material or, more usefully, through a repository. Repositories are large data storage servers with a website front-end that can be used to upload and share data publicly (e.g. Dryad [17], FigShare [18], OpenScience framework [19], or Zenodo [20]). A license allowing modification and resharing should be attached to these data to maximize the speed of research discoveries.

### Document, manage and save data analysis batch scripts and workflows

#### Making analyses reproducible with limited programming skills

Functional MRI analyses are complex, involving many pre-processing steps as well as a multitude of possible statistical analyses. Even if the most important steps are reported using precise guidelines [21], there are too many parameters involved in the data analysis process to be able to provide a full description in any article. Carp [7] examined a simple event-related design using common neuroimaging tools, but varying the available settings (see also [8]). This led to 6,912 unique analysis pipelines, and revealed that some analysis decisions contributed to variability in activation strength, location and extent, and ultimately to inflated false positive rates [4]. In the face of such variability, some have argued that ‘anything less than release of actual source code is an indefensible approach for any scientific results that depend on computation, because not releasing such code raises needless, and needlessly confusing, roadblocks to reproducibility’ [22].

In contrast with data analysts or software developers, many neuroimagers do not code their analysis from scratch - instead they rely on existing software and often reuse code gathered from others in the laboratory or on the web. Pressing buttons in a graphical user interface is not something that can be replicated, unless inputs and processing steps are saved in log files. To ensure reproducibility (even for oneself in a few months’ time) one needs to set up an automatic workflow. Informatics and bioinformatics researchers have been discussing issues of code reproducibility for many years [23,24], and lessons can be learnt from their experience. Sandve et al. [24] have a few simple recommendations. First, keep track of every step, from data collection to results, and whenever possible keep track with electronic records. Most neuroimaging software has a so-called batch mode (SPM [25,26]) or pipeline engine (Nipype [27,28]), or is made up of scripts (AFNI [29,30], FSL [31,32]), and saving these is the best way to ensure that one can replicate the analysis. At each step, record electronically, and if possible automatically, what was done with what software (and its version). Second, minimize, and if possible eliminate, manual editing. For instance, if one needs to convert between file formats, this is better done automatically with a script, and this script should be saved. Third, for analyses that involve a random number generator, save the seed or state of the system, so that the exact same result can be obtained. As for the computer program used to run the experiment (step 1), the batch and scripts can be made available as supplementary material in a journal, and/or shared in repositories. If one ends up with a fully functional script that includes a new type of analysis, this can itself be registered as a tool on dedicated websites such as the NeuroImaging Tool and Resources Clearinghouse (NITRC [33]). Sharing the analysis batch and scripts is the only way to ensure reproducibility by allowing anyone to (i) check for potential errors that ‘creep in’ to any analyses [10]; (ii) reuse them on new data, possibly changing a few parameters to suit changes in scanning protocol - similar results should be observed if the effects were true [14] - and (iii) base new analysis techniques or further research on verifiable code.

#### Improving scripts and turning them into workflows

Although these recommendations are, we hope, useful, they are not generally sufficient. Analysis code depends on software, operating systems, and libraries that are regularly updated (see, e.g. [34] for an effect on imaging results). When the code is rerun, these changes should be tracked, and results attached to a specific version of the code and its environment. The only complete solution is to set up virtual machine or equivalent. For neuroimaging, the NeuroDebian project [35] integrates relevant software into the Debian operating system, where all software is unambiguously versioned and seamlessly available from a package repository. This makes it possible to define the whole environment and reconstruct it at any later time using snapshots of the Debian archive [36]. While such a solution is the most complete, investing in good revision control software is a first step that goes a long way in handling code (Wikipedia lists 36 types of such software [37]). We argue here that this investment is a necessity for reproducible science.

Although a simple text editor or word processing document could be used to precisely describe each analysis step, only an executable script and information on the associated software environment can give one a reasonable chance of reproducing an entire experiment. This implies that much more should be done to teach programming to students or researchers who need to work with neuroimaging data. Barriers to code sharing are not as great as for data, but they do exist. Researchers are often concerned that their code is too poor, and that there might be some errors. These, and the fear of being ‘scooped’, are some of the main reasons scientists give for not sharing code with others [38]. Yet, as Barnes [39] puts it, “software in all trades is written to be good enough for the job intended. So if your code is good enough to do the job, then it is good enough to release”. A few simple rules can be applied to improve scripts [23]. First, make your code understandable to others (and yourself). Add comments to scripts, providing information not just about what is computed, but also reflecting what hypothesis is being tested, or question answered, by that specific piece of code [24]. Second, version control everything. Version control systems (VCSs) store and back up every previous version of the code, allowing one to ‘roll back’ to an older version of the code when things go wrong. Two of the most popular VCSs are Git [40] (which we recommend) and Subversion [41]. ‘Social coding’ platforms, such as GitHub [42] or Bitbucket [43], are also useful sharing and collaboration tools. Third, test your code effectively, to assure yourself and others that it does what it is supposed to. The software industry tells us that “untested code is broken code”, but scientists lack incentives to invest time in this. For example, if you coded some statistical tests to be run on multiple voxels, compare the routine in one voxel against a prototype solution. Learning how to test and document one’s code is a crucial skill to reduce bugs and ensure safe reuse of code, an aspect that is not sufficiently emphasized and taught in curricula. In fact, the experience of the authors is that it is hardly ever mentioned.

Neuroimagers can also take advantage of a few easy-to-use tools to create complex scripts and make a workflow (a workflow consists of a repeatable pattern of activities that transform data and can be depicted as a sequence of operations, declared as work of a person or group (adapted from [44]). For Matlab-based analyses, we can recommend using Matlab-specific formatting^a^ in the code, and a workflow engine such as the Pipeline System for Octave and Matlab (PSOM [45,46]) or the Automatic Analysis pipeline (AA [47,48]). For Python-based analyses, we recommend the IPython notebook ([49] now the Jupyter project) to sketch the analysis and explore results, along with the workflows provided in Nipype [27,28]. Packages such as SPM [25,26] have batch systems that create scripts of the whole analysis workflow, which should be learned for efficiency, reproducibility and provenance tracking. It is also possible to create entire workflows using general (e.g. Taverna [50], Kepler [51]) or dedicated libraries (LONI pipeline [52]) and thereby obtain analysis provenance information. Using these pipelines, one can create (via a graphical interface or a script) a workflow of the different steps involved in fMRI data processing, specifying parameters needed at each step, and save the workflow. Dedicated libraries or scripts can be called, and the impact of changing a parameter value in a specific implementation of a step can be studied. Most of these pipeline systems have ways to help distribute the processing using computers’ multicore architectures, or job-scheduling systems installed on clusters, thereby reducing computation time. In general, these tools require some programming and software expertise (local installation and configuration issues seem to be largely underestimated issues) beyond what fMRI researchers can usually do (whereas PSOM, Nipype and using the SPM batch system are ‘easy’). These more complex workflow or pipeline solutions can, however, ease replication of the analysis by others: see [53] for an example using the LONI pipeline.

### Organize and share data and metadata

Besides replicating an analysis (running exactly the same code on the same data), sharing data provides guarantees of reproducibility by (i) allowing a comparison with newly collected data (are the patterns observed in the new dataset the same, independently of statistical significance?), (ii) allowing alternative analyses to be tested on the same data, and (iii) aggregating them with other data for meta-analyses [54]. Many funders now request that data are made available, and researchers must be prepared to do this and to identify where the data will be archived. When the data have obvious potential for reuse (e.g. [55]) or pose special challenges (e.g. [56]), their publication in journals such as *Data in Brief, Frontiers in Neuroscience, F1000 Research, GigaScience*, *Journal of Open Psychology Data*, or *Scientific Data* allow the creators to be acknowledged by citation. In any case, data can simply be put in a repository such as NITRC [33] or Open-fMRI [57] (task-based fMRI [58]). As of March 2015, OpenfMRI hosts 33 full datasets, and a more complete format describing the data is being developed. Previously, the major project that supported sharing of full fMRI datasets was the fMRI Data Center [59,60]. It currently has 107 datasets available on request, but has not accepted submission of additional datasets since 2007. The researcher must also be aware of the constraints involved in sharing MRI data. It is of course essential that consent forms indicate clearly that the data will be de-identified and shared anonymously, and it is the responsibility of the principal investigator to ensure proper de-identification [61], that is, not only removing any personal information from the image headers, but also removing facial (and possibly dental and ear) information from the T1-weighted image. Fortunately, personal information is removed automatically by most fMRI packages when converting from DICOM to NIfTI file format. Removing facial information can be trickier, but automated tools exist for this too (SPM [25,26], MBRIN defacer [62,63], Open fMRI face removal Python script^b^).

Another important issue to consider when sharing data is the metadata (information describing the data). Data reuse is only practical and efficient when data, metadata, and information about the process of generating the data are all provided [64]. Ideally, we would like all of the information about how the data came to existence (why and how) to be provided. The World Wide Web Consortium Provenance Group [65] defines information ‘provenance’ as the sum of all of the processes, people (institutions or agents), and documents (data included) that were involved in generating or otherwise influencing or delivering a piece of information. For fMRI data, this means that raw data would need to be available, along with (i) initial project information and hypotheses leading to the acquired data, including scientific background as well as people and funders involved; (ii) experimental protocol and acquisition details; and (iii) other subject information, such as demographics and behavioral or clinical assessments. There are currently no tools to do this metatagging, but we recommend checking with the database that will host the data and using their format from the start (that is, store data on your computer or server using the same structure). Functional MRI can have a complex data structure, and reorganizing the data post-hoc can be time-consuming (several hours for posting on OpenfMRI, if the reorganization is done manually [66]). In the future, efforts spearheaded by the International Neuroinformatics Coordinating Facility (INCF [67]) data sharing task force (INCF-Nidash [68]) may provide a solution, with the development of the Neuro-Imaging Data Model (NIDM [69]), as well as some recommendations on the directory structure and metadata to be attached to the data. Some initial work already permits meta-information to be attached directly to SPM [25,26], FSL [31,32], and (soon) AFNI [29,30] fMRI data analysis results.

### Make derived data available

Along with the raw data and the analysis batch and scripts, sharing derived data also increases reproducibility by allowing researchers to compare their results directly. Three types of derived data can be identified: intermediate derived data (from the data analysis workflow), primary derived data (results) and secondary derived data (summary measurements).

Providing intermediate derived data from the analysis workflow, such as the averaged echo-planar image (mean EPI) or statistical mask, makes it possible to judge whether an analysis provides reasonable-looking data, and what the residual brain coverage is after realignment, normalization and subject overlay. Intermediate derived data may not always be directly essential to reproducibility, but can improve the confidence in the data at hand and/or point to their limitations. More important for reproducibility is the sharing of primary derived data. Currently, fMRI studies only report significant results (regions that survive the statistical threshold), because one cannot list all regions or voxels tested. Yet results are more often reproduced when reported at a less conservative significance threshold (p-value) than is often used in our community [70]. The best way to validate that an experiment has been reproduced is by comparing effect sizes, independently of the significance level. Comparing peak coordinates of significant results can be useful, but is limited [66]. In contrast, providing statistical or parameter maps allows others to judge the significance and sparsity of activation clusters [71]. Statistical maps can be shared via NeuroVault [72,73]. NeuroVault allows the visualization and exploration of raw statistical maps and is thus a good way look not only at effect sizes, but also at the precise location of effects (rather than the crude cluster peak coordinate). Along with the statistical maps, some information about provenance currently has to be entered manually (taking 10 to 15 minutes). Again, this manual editing will soon be facilitated by the adoption of the NIDM [69]. Finally, as for statistical maps, secondary derived data should be shared - most likely as supplementary material data sheets. In a region of interest (ROI) analysis, for instance, the mean parameter values extracted across voxels are assembled into a matrix to compute statistics. This data matrix should be saved and distributed so that effect sizes can be compared across studies. Providing scatter plots along with the data of any zero-order, partial, or part correlations between brain activity or structure and behavioral measures also allows one to judge of the robustness of the results [74].

### Publish

One aspect to consider when sharing data is to make them available online before publication, so that permanent links can be included in the article at the time of publication. We also recommend stating how you want data and code to be credited by using machine-readable licenses. Easy-to-implement licenses, many of which offer the advantage of being machine-readable, are offered by the Creative Commons organization [75] and Open Data Commons [76].

## Discussion

Researchers are much more likely to be able to replicate experiments and reproduce results if material and procedures are shared, from the planning of an experiment to the fMRI result maps. This is also crucial if the global efficiency of our research field is to improve. To be able to do this, the single most important advice to consider would probably be to plan ahead, as lack of planning often prevents sharing^c^. Informed consent and ethics should be compliant with data sharing. When previous data are available, statistical power should be computed, sample size chosen accordingly and reported. Data, scripts and maps should be organized and written with the intention to share and allow reuse, and they should have licenses allowing redistribution.

To increase fMRI reproducibility, neuroscientists need to be trained, and to train others, to plan, document and code in a much more systematic manner than is currently done. Neuroimaging is a computational data science, and most biologists, medical doctors and psychologists lack appropriate programming, software and data science training. In that respect, sharing work has an additional educational value. By studying the code used by others, in order to replicate their results, one also learns what practices are useful when sharing. Piwowar et al. [77] showed that sharing data and code increases the trust and interest in papers, and citation of them. This also makes new collaborations possible more easily. Openness improves both the code used by scientists and the ability of the public to engage with their work [39]. Putting the code associated with a paper in a repository is likely to have as many benefits as sharing data or publications. For instance, the practice of self-archiving can increase citation impact by a dramatic 50 to 250% [78]. Data and code sharing can also be viewed as a more ethical and efficient use of public funding (as data acquired by public funds should be available to the scientific community at large), as well as a much more efficient way of conducting science, by increasing the reuse of research products.

## Conclusion

By adopting a new set of practices and by increasing the computational expertise of fMRI researchers, the reproducibility and validity of the field’s results will improve. This calls for a much more open scientific attitude in fMRI, together with increased responsibility. This will advance our field more rapidly and yield a higher return on funding investment. Making neuroimaging reproducible will not make studies better; it will make scientific conclusions more verifiable, by accumulating evidence through replication, and ultimately make those conclusions more valid and research more efficient. Two of the main obstacles on this road are the lack of programming expertise in many neuroscience or clinical research laboratories, and the absence of widespread acknowledgement that neuroimaging is (also) a computational science.

## Annex 1 - list of websites mentioned in the article that can be used for sharing

**Bitbucket** (https://bitbucket.org/) is “a web-based hosting service for projects that use either the Mercurial or Git revision control system” and allows managing and sharing code.

**Dryad** (http://datadryad.org/) “is a curated resource that makes the data underlying scientific publications discoverable, freely reusable, and citable” under a Creative Commons license. It is a nonprofit membership organization from an initiative among a group of leading journals and scientific societies in evolutionary biology and ecology. This repository now hosts any kind of biological data.

**FigShare** (http://figshare.com/) is a repository that “allows researchers to publish all of their data in a citable, searchable and sharable manner” under a Creative Commons license. It is supported by Digital Science, part of Macmillan Publishers Limited. This repository now hosts any kind of data.

**GitHub** (https://github.com/) is “a web-based Git repository hosting service” and allows managing and sharing code.

**Kepler** (https://kepler-project.org/) is a scientific workflow application “designed to help scientists, analysts, and computer programmers create, execute, and share models and analyses across a broad range of scientific and engineering disciplines”.

**LONI pipeline** (http://pipeline.bmap.ucla.edu/) is an application to “create workflows that take advantage of all the tools available in neuroimaging, genomics [and] bioinformatics”.

**NeuroDebian** (http://neuro.debian.net/) integrates neuroimaging and other related neuroscientific and computational software into Debian (Linux). It includes a repository of over 60 software and data packages. NeuroDebian also provides a virtual machine, simplifying deployment within any existing Linux, OS X or Windows environment.

**NeuroImaging Tool and Resources Clearinghouse** (http://www.nitrc.org/), is a web resource that “facilitates finding and comparing neuroimaging resources for functional and structural neuroimaging analyses”. It is currently funded by the NIH Blueprint for Neuroscience Research, National Institute of Biomedical Imaging and Bioengineering, National Institute of Drug Addiction, National Institute of Mental Health, and National Institute of Neurological Disorders and Stroke.

**NeuroVault** (http://neurovault.org/) is a “public repository of unthresholded brain activation maps” under a data common license. It is managed by Krzysztof Gorgolewski, and supported by INCF and the Max Planck Society.

**Open fMRI** (https://openfmri.org/) is “a project dedicated to the free and open sharing of functional magnetic resonance imaging (fMRI) datasets, including raw data” under an open data common license. It is managed by Russ Poldrack and funded by a grant from the National Science Foundation.

**OpenScience framework** (https://osf.io/) is a project management system for an “entire research lifecycle: planning, execution, reporting, archiving, and discovery”. It supports local archiving, but also links with other repositories. Multiple options for licensing are available. It is supported by the Center for Open Science.

**Taverna** (http://www.taverna.org.uk/) is a “domain-independent workflow management system - a suite of tools used to design and execute scientific workflows”.

**Zenodo** (http://zenodo.org/) is a repository “that enables researchers, scientists, EU projects and institutions to share and showcase multidisciplinary research results”, with a choice of open source licenses. It was launched within an EU funded project and is supported by the European Organization for Nuclear Research (CERN).

## Endnotes

^a^Matlab Publishing Markup refers to specific keys such as %% or _ _ which allows not only inserting comments into your Matlab code, but also format it for then publish the code automatically into an executable and readable format, see http://uk.mathworks.com/help/matlab/matlab_prog/marking-up-matlab-comments-for-publishing.html.

^b^When uploading data to OpenfMRI you need to ensure the structural data are defaced appropriately – the website also offers to use their own defacing tool, see https://github.com/poldrack/openfmri/tree/master/pipeline/facemask.

^c^Thanks to Dorothy Bishop for pointing to this.

## Abbreviations

AFNI: Analysis of functional neuroimages
fMRI: Functional magnetic resonance imaging
FSL: FMRIB software library
INCF: International neuroinformatics coordinating facility
NIDM: Neuro-imaging data model
Nipype: NeuroImaging in python pipelines and interfaces
PSOM: Pipeline system for octave and matlab
SPM: Statistical parametric mapping
